# Direct and indirect interactions of the dopamine D_3_ receptor with glutamate pathways: implications for the treatment of schizophrenia

**DOI:** 10.1007/s00210-012-0797-0

**Published:** 2012-09-22

**Authors:** Pierre Sokoloff, Ludovic Leriche, Jorge Diaz, Jacques Louvel, René Pumain

**Affiliations:** 1 Pierre Fabre Research Center, Castres, 81100 France; 2 Neurobiology and Pharmacology (INSERM U573), Paris, 74014 France; 3 Laboratoire de Physiology, Université René Descartes Paris V, Paris, 75005 France; 4 Psychiatry and Neuroscience Center (INSERM U894), Paris, 75014 France

**Keywords:** Asymmetric synapse, VGluT1, BP897, Prefrontal cortex, *c-fos*, Paired-pulse facilitation, F17141, Social interaction deficit

## Abstract

This article, based on original data as well as on previously reported preclinical and clinical data that are reviewed, describes direct and indirect interactions of the D_3_ receptor with *N*-methyl-d-aspartate receptor (NMDA) signaling and their functional consequences and therapeutic implications for schizophrenia. D_3_ receptor immunoreactivity at ultrastructural level with electron microscopy was identified at presumably glutamatergic, asymmetric synapses of the medium-sized spiny neurons of the nucleus accumbens. This finding supports the existence of a direct interaction of the D_3_ receptor with glutamate, in line with previously described interactions with NMDA signaling involving Ca^2+^/calmodulin-dependent protein kinase II at post-synaptic densities (Liu et al. 2009). Indirect interactions of the D_3_ receptor with glutamate could involve a negative control exerted by the D_3_ receptor on mesocortical dopamine neurons and the complex regulation of the glutamatergic pyramidal cells by dopamine in the prefrontal cortex. This could be exemplified here by the regulation of pyramidal cell activity in conditions of chronic NMDA receptor blockade with dizocilpine (MK-801). BP897, a D_3_ receptor-selective partial agonist, reversed the dysregulation of cortical *c-fos* mRNA expression and pyramidal cell hyperexcitability, as measured by paired-pulse electrophysiology. At the behavioral level, blockade of the D_3_ receptor, by known D_3_ receptor antagonists or the novel D_3_ receptor-selective antagonist F17141, produces antipsychotic-like effects in reversing hyperactivity and social interaction deficits induced by NMDA receptor blockade by MK-801 in mice. The glutamate–D_3_ receptor interactions described here offer a conceptual framework for developing new D_3_ receptor-selective drugs, which may appear as an original, efficacious, and safe way to potentially indirectly target glutamate in schizophrenia.

## Introduction

Since its discovery (Sokoloff et al. 1990), much progress has been accomplished toward understanding the physiological role of the dopamine D_3_ receptor and defining the potential indications of pharmacological drugs specifically acting on this receptor. At the anatomical level, the brain regions where the D_3_ receptor is expressed have been delineated, and the phenotypes of D_3_ receptor-expressing neurons have been characterized (Bouthenet et al. 1991; Diaz et al. 1995). These studies underscore the limited and restricted distribution of the D_3_ receptor in the brain, seemingly related to functions of dopamine associated with the limbic brain. Hence, the hypothesis has been put forward that the D_3_ receptor could be involved in the pathophysiology of several psychiatric disorders, which result from dysfunction of dopamine neurotransmission (Sokoloff et al. 2006).

Several review articles published during the last decade, as well as earlier and recent original research articles, have been published to support this hypothesis. Nowadays, compelling evidence from preclinical and genetic studies exists to implicate the D_3_ receptor in three major pathological domains, namely, drug addiction, movement disorders, and schizophrenia, even though only little clinical results came in support. In this respect, a major advance has been accomplished with the discovery and use of D_3_ receptor-selective pharmacological drugs. Starting with the phenylpiperazine derivative BP 897 (Pilla et al. 1999), a number of original compounds, yet with structural similarities, were designed (Micheli and Heidbreder 2006; Boeckler and Gmeiner 2006), and a patent survey indicates that, since 2005, 110 patents or patent applications have been published, which shows that this is still a very active research area, in both academic and industrial laboratories. In these patents, a number of indications are targeted by D_3_ receptor-selective drugs. However, those for which the rationale is supported by compelling data are more limited, and they will be briefly reviewed here; the reader is also invited to refer to accompanying articles of this special issue for an updated information.

Drug addiction remains one of the first indications for D_3_ receptor partial agonists and antagonists. Following the discovery of the role of D_3_ receptor in cocaine self-administration (Caine and Koob 1993) and in cue-induced cocaine-seeking behavior (Pilla et al. 1999), numerous studies have investigated D_3_ receptor drugs in animal models of addiction to various abused drugs, including psychostimulants, opioids, nicotine, and alcohol. The preclinical evidence indicates that the D_3_ receptor antagonism diminishes motivation to self-administer drugs, in particular in conditions of high price for drugs, and disrupts drug-associated cue-induced craving and stress-induced reinstatement (Heidbreder et al. 2005; Heidbreder and Newman 2010; Le Foll et al. 2005; 2007). The modulation by the D_3_ receptor of the effects of food-associated cues on overweight and obese patients has recently been demonstrated in a clinical study (Nathan et al. 2012).

Movement disorders include several conditions, of which a detectable increased risk could be associated with the presence of the Gly allele of *DRD3* polymorphism, which showed an increased function in vitro compared to the Ser allele (Jeanneteau et al. 2006). The movement disorders include tardive dyskinesia (Lerer et al. 2002), a debilitating and stigmatizing condition emerging after long-term treatment with antipsychotics and essential tremor (Jeanneteau et al. 2006), a slowly progressive disorder most frequently characterized by an action (kinetic or postural) tremor of the arms and hands. Restless legs syndrome involves abnormal limb sensations that diminish with motor activity and exacerbate at night. It has been associated with the D_3_ receptor, on the basis that the therapeutic agents for this condition are D_3_-preferential agonists. Additionally, D_3_ receptor-deficient mice exhibit facilitation rather than the expected depression of spinal reflexes in the presence of dopamine (Clemens et al. 2006) and also resemble a mouse model of restless legs syndrome, with respect to sensory and motor symptoms (Dowling et al. 2011). Dyskinesia in Parkinson’s disease consists of intractable and pharmacoresistant involuntary movements resulting to sensitization to substitution treatment, particularly l-DOPA, and has been associated in rat (Bordet et al. 1997) and monkey (Bezard et al. 2003) models with an increased D_3_ receptor expression and function (see also Visanji et al. 2006).

The rationale of using D_3_ receptor antagonists in the treatment of schizophrenia mainly arises from the observation that all registered antipsychotic drugs bind with almost equal affinities to D_2_ and D_3_ receptors in vitro (Sokoloff et al. 1992; Malmberg and Mohell 1995). Whether these drugs actually bind to cerebral D_3_ receptors at therapeutically active doses has been a matter of controversy. In a positron emission tomography (PET) study in patients with schizophrenia, atypical antipsychotics failed to occupy D_3_ receptors in the D_3_-rich brain regions globus pallidus and substantia nigra (Graff-Guerrero et al. 2009a; Mizrahi et al. 2011). However, a PET study in non-human primates issued from another laboratory (Girgis et al. 2011) provided evidence that acute therapeutically active doses of clozapine and haloperidol readily bind to D_3_ receptors in vivo and that the discrepancies with the former studies can be accounted by methodological considerations. Besides this pharmacological evidence, there are also compelling data, which will be reviewed in detail below, showing that the D_3_ receptor is localized at positions in neurons critical for controlling psychotic symptoms and that preclinical models of schizophrenia reveal the antipsychotic-like properties of D_3_ receptor antagonists, of which one original compound will be described.

Our major aims in this article will be to extract from the above literature, to present novel experimental evidence, and to discuss the hypothesis that the physiological role of the D_3_ receptor in schizophrenia may actually be underlined by direct and indirect interactions of this receptor with glutamate pathways. This hypothesis is consistent with the idea that schizophrenia results from both dopamine and glutamate dysfunctions and from dopamine–glutamate imbalance (Carlsson 1988; Goff and Coyle 2001; Javitt 2004). It may also offer a theoretical frame for the use of D_3_ antagonists in the treatment of schizophrenia, as a therapeutic alternative to direct glutamatergic antipsychotic drugs, such as agonists or positive modulators of the metabotropic glutamate receptor subtypes 2 and 3 (mGluR2/3) (Patil et al. 2007) and inhibitors of type 1 glutamate uptake (Alberati et al. 2012), which are presently promising treatments, but, until now, have not been consistently proved to be efficacious and safe.

## Methods

### Drugs

(+)-Dizocilpine maleate (MK-801, Sigma), BP 897 (dihydrochloride salt, Bioprojet), and F17141 (hydrochloride salt, synthesized at Pierre Fabre Research Institute) were dissolved in sterile water. All solutions will be prepared fresh daily and injected in a volume of 10 ml kg^-1^. Doses are expressed in milligram per kilogram of the free base.

### Animals

For immunochemistry, rats were anesthetized deeply with sodium pentobarbital and then perfused transcardially with saline solution (50 ml of 0.9 % NaCl warmed at 37 °C), followed by 600 ml of an ice-cooled fixative solution containing 3 % paraformaldehyde in 0.1 M phosphate buffer, pH 7.4, or containing 3 % paraformaldehyde, 0.1 % glutaraldehyde, 0.2 % picric acid in 0.1 M PB, pH 7.4, for immunoelectron microscopy. The brains were removed and post-fixed for 2 h at 4 °C in the same fixative used for perfusion. Brains were cut with a vibratome in coronal sections of 40 μm that were cryoprotected in 0.1 M phosphate buffer, pH 7.4, containing 30 % sucrose and freeze-thawed (−75 °C) before use for immunostaining.

For behavioral studies, male Swiss mice Crl:OF1 (IFFA CREDO, France), weighting 20–22 g upon arrival, were housed five to eight per cage with sawdust bedding (polycarbonate type III cages; *L* 375 mm, *l* 215 mm, *H* 149 mm; floor surface 806 cm^2^), on a 12-/12-h light/dark cycle (lights on at 07:00 a.m.), with food and water freely available. Room temperature ranged from 20 to 22 °C, and humidity varied between 55 and 65 %. In the resident intruder test, half of the mice were single-housed (Cristal PS cages , L 220 mm, 85 mm, H 80 mm; floor surface 187 cm^2^) until the end of the experiment (see below).

### Immunohistochemistry

For double-immunofluorescent labeling, cryostat sections (10 μm) of rat brain nucleus accumbens were blocked for 1 h at room temperature in 0.05 M Tris-buffered saline (TBS), pH 7.4, containing 5 % donkey normal serum, 0.4 % BSA, 0.1 % gelatin, and 0.1 % Tween-20. Sections were incubated (48 h at 4 °C) with the rabbit anti-D_3_R antibody (1:2,000) in combination with the guinea pig antibody to the vesicular glutamate transporter 1 (VGluT1, 1:5,000; Chemicon, Temecula, CA, USA) or the antibody to vesicular glutamate transporter 2 (VGluT2, 1:2500; Chemicon, Temecula, CA), in TBS containing 5 % normal donkey serum and 0.05 % Tween-20. After four washes in TBS, 0.05 % Tween-20, sections were incubated for 1 h at room temperature with the antibodies donkey anti-rabbit-CY3 (1:200) and the donkey anti-guinea pig-Alexa-488 (1:200) (Molecular Probes, Corisbad, CA, USA) in TBS 0.05 %, Tween-20. Sections were washed, mounted on Super Frost Plus slides, and then coverslipped using Mowiol. Control experiments were performed to ensure that each primary antibody did not react with the non-corresponding secondary antibody conjugate. Immunostained sections were analyzed and photographed on a Zeiss Axiophot microscope.

### Immunoelectron microscopy

Nucleus accumbens sections of three rats were blocked for 1 h (as for immunohistochemistry) and then incubated 72 h at 4 °C with the rabbit anti-D_3_R antibody (1:2,000) in 0.05 M TBS, pH 7.4, containing 10 % normal donkey serum. Some sections were incubated without the antibody and used as control of specific immunostaining. After four washes in 0.05 M TBS, pH 7.4, containing 0.1 % gelatin, sections were incubated for 1 h at room temperature with biotinylated donkey anti-rabbit IgG (1:200; Amersham) in 0.05 M TBS, pH 7.4. Following rinsing (three times for 10 min) in 0.05 M TBS, pH 7.4, 0.1 % gelatin, sections were incubated for 1 h at room temperature in avidin–biotin–HRP complex (ABC reagent, Vectastain Elite; Vector Laboratories, Burlingame, CA, USA). After peroxidase immunostaining, sections were rinsed in 0.05 M PBS, pH 7.4; fixed for 10 min in 1 % glutaraldehyde, 0.05 M PBS, pH 7.4; and then post-fixed for 20 min in 1 % osmium tetroxide. Brain sections were dehydrated in a graded series of ethanol solutions, impregnated with 1 % uranyl acetate in 100 % alcohol, and infiltrated and flat-embedded in polymerized epoxy resin. Pieces of epone-embedded sections were then cut from the accumbens and glued to carrier blocks, and ultrathin sections were cut from these specimens with a Reichert ultramicrotome. Ultrathin sections were mounted on mesh grids, stained with 0.4 % lead citrate and 4.0 % uranyl acetate, and finally analyzed and photographed on a JEOL 100 electron microscope.

### Treatments for chronic NMDA receptor blockade

For chronic NMDA receptor blockade, dizocilpine was continuously infused for 7 days via osmotic minipumps (Model 1007D, Alzet Corp.) from day 0 to day 7. Minipumps were filled 6 h prior to implantation and placed in a sterile saline water bath at 37 °C. Taking into account the average mouse weight at the time of testing and the delivery rate of minipumps (specified by the manufacturer), we adjusted the MK-801 concentration for a delivery of 0.02 mg kg^-1^ h^-1^. Mice were anesthetized with chloral hydrate (400 mg kg^-1^ i.p., Centravet, France) or isoflurane (O_2_ flow rate 2–3 l min^-1^, induction 3.5 %, and maintenance 1.5–2.5 % isoflurane). Mice were placed on a thermostatically controlled heating pad connected to a rectal probe to maintain the animals in homeothermic condition (37 °C) during surgery. An area on the back of the mice was shaved and disinfected thoroughly using Vétédine® (Centravet, France). A 1-cm incision was made in the skin between the scapulae. Using a hemostat, a small pocket was formed by spreading apart the subcutaneous connective tissues. The pump was inserted into the pocket, and the skin incision was closed with metallic wound clips. The skin was again disinfected with Vétédine®, and Négérol® spray (Centravet, France) was sprayed on the wound. Mice were returned in their home cage, and in about 2 h (chloral hydrate anesthesia) or 10 min (isoflurane anesthesia), they had fully recovered and were active.

### *c-fos* in situ hybridization

Saline or BP 897 (1 mg kg^-1^ i.p.) was given twice daily for 4 days beginning 5.5 days after minipump implantation (from day 5.5 to end of day 9), and animals were then sacrificed on day 10 at least 14 h after the last injection, when no more acute effect of drugs where observed. Their brains were frozen in isopentane (−30 °C) and stored at −80 °C until used. Slices (10 μm) were hybridized with a ^33^P-labeled antisense cRNA probe for *c-fos* mRNA. For *c-fos* mRNA quantification, autoradiographic signals were quantified on three to six slices per animal using an image analyzer (IMSTAR, France). Gray values were converted to microcurie per milligram dry weight using C14 standard stripes (Amersham). For c-fos mRNA imaging, slices were hybridized with a [^33^P]-labeled *c-fos* cRNA probe and a digoxigenin-labeled *VGluT1* cRNA probe (the plasmid containing cDNA encoding VGluT1 was generously donated by S. El Mestikawy). The hybridized slices were dipped into the photographic emulsion, and silver grain density was assessed in discrete neuronal populations using “Grain®” imaging software (IMSTAR, France). Briefly, silver grains were counted over randomly selected VGluT1-labeled neurons from the prefrontal cortex for each animal (60–80 cells) in three separate sections.

### Electrophysiological recordings

Saline or BP 897 (1 mg kg^-1^ i.p.) was given twice daily for 4 days beginning 5.5 days after minipump implantation (from day 5.5 to end of day 9), and animals were then sacrificed on day 10 at least 14 h after the last injection, when no more acute effects of drugs were observed. At day 10, mice were sacrificed, and their brains were rapidly removed and transferred to a chilled (4 °C) slicing solution equilibrated with a 95:5 % mixture of O_2_ and CO_2_. Coronal slices (300 μm thick) were prepared on a vibratome (blade angle, 18°) and transferred to a humidified interface chamber at 33 °C. The slices were superfused with oxygenated artificial cerebrospinal fluid (ACSF: 126 mM NaCl, 26 mM NaHCO_3_, 3 mM KCl, 1.25 mM NaH_2_PO_4_, 2 mM MgCl_2_, 2 mM CaCl_2_, and 10 mM glucose; pH 7.4) at a rate of 2.5 ml min^-1^ for 90 min before recordings began. There was usually one slice in each hemisphere with the cortex of interest. Extracellular field potentials were recorded using borosilicate glass microelectrodes filled with ACSF (1–2 MΩ). A microelectrode was placed in the middle layers of the prelimbic cortex, and a bipolar stainless steel stimulating electrode was placed in the underlying white matter. Electrodes were positioned to provide the lowest stimulation threshold to evoke a response and the smallest possible stimulation artifact. For a given configuration, the threshold for triggering a response was determined using single shocks (80-μs square direct current (DC) pulses), and the stimulus intensity was then set at three times this threshold for the rest of the experiment. A common stimulation sequence was programmed using the Spike2® programming language. The stimulation sequence began with and ended with a series of 12 single shocks delivered every 5 s; the initial single-shock series was followed, after a 30-s silent period, by a series of paired stimuli delivered every 10 s. Each pair of stimuli had an interpulse interval that was shortened by half that of the preceding one starting from 1,000 ms down to 7.8 ms. The sequence with varying interpulse intervals was repeated eight times.

The signal was amplified (×1,000) and filtered (DC 3 kHz, analog filter) before being digitized (10 kHz) using a CED 1401 interface and the Spike2® software (Cambridge Electronic Design, UK). In an off-line analysis, the 12 single-shock responses and the eight pairs of responses to paired shocks with the same interpulse interval were averaged for visual inspection and comparison. For each response, the amplitude was computed by a homemade program as the difference between the maximum potential value and the minimum potential value in a time range that was predetermined by visual inspection. For each pair of stimuli, the ratio of the second response amplitude to the first one was computed to normalize the measurements. Again, the resulting ratio for the eight pairs of responses to paired shocks with the same interpulse interval was then averaged.

### Locomotor activity after acute NMDA receptor blockade

The procedure has been described previously (Leriche et al. 2003) with small adaptations. Briefly, mice were injected with either saline or F17141 at doses of 0.16, 0.63, 2.5, or 10 mg kg^-1^ i.p., and their horizontal locomotor activity was measured for 30 min (spontaneous locomotor activity). They were subsequently treated with 0.9 % NaCl (saline) in a volume of 10 ml kg^-1^ or dizocilpine (0.14 mg kg^-1^ i.p.), and the activity was measured for 1 h (MK-801-induced locomotor activity). The locomotor activity was measured in an actimeter that was composed of eight individual activity cages (30 × 15 × 18 cm, with sawdust on the floor) transected by infrared beams (Imetronic, Pessac, France). Counts for forward horizontal activity were incremented each time the animal moved from one-half part of the cage to the other, corresponding to disruption of two crossed parallel beams distant of 14 cm.

### Resident–intruder assay after chronic NMDA receptor blockade

Male mice were housed individually (residents) or in groups of five (intruders) for 1 week prior to minipump implantation (see above), delivering MK-801 (0.02 mg kg^-1^ h^-1^ s.c.) or saline (Sham) as described above. Following recovery, they were housed again in the same condition for 7 days and then tested in a “resident–intruder” paradigm adapted from (Dixon et al. 1994; Mohn et al. 1999) with some modifications. In the original test, a resident mouse is housed alone for 2 weeks, and then, a group-housed intruder is introduced to the resident cage. In our experiments, for convenience of observation, group-housed intruder and isolated resident were not confronted in the resident cage but in a test arena (34 cm × 34 cm × 30 cm, *l* × *L* × *h*) with sawdust on the floor. Sawdust will be replaced between each habituation or test session. At the end of a day of experiments, the arena will be cleaned using 70 % EtOH. Even with these modifications, the behaviors displayed by both group of mice were almost the same as those observed in the original conditions cited above (Leriche, personal observations; see “Results and discussion”). To minimize the incidence of stress on social behavior, mice were habituated the day before the test day 6 min to the test arena. In these conditions, placing the animals in test arena was as less aversive as possible.

The day before the resident–intruder social interaction test, all the mice from the intruder groups were labeled with a different color code with a permanent marker to differentiate them during the test session. Thirty minutes prior to testing, saline, F17141 (0.16, 0.63, 2.5, or 10 mg kg^-1^), or clozapine (1 mg kg^-1^) were administered to the resident and intruder mice. As described previously, a group-housed male (intruder) and an individually housed male (resident) from the same treatment group (i.e., saline-resident and saline-intruder, etc.) were introduced in the test arena, and their behaviors were video-recorded for 6 min. The videotaped behaviors of the resident and intruder mice were individually scored for time spent in social investigation (approaching, sniffing, grooming other mouse, and sexual behavior), in escape behavior (actively avoiding the unfamiliar congener), and fighting. Typically, the resident actively initiates social investigations of the intruder, sometimes initiating fights, and rarely avoids social interaction by escape (Dixon et al. 1994; Mohn et al. 1999).

### Statistical analysis

Between-groups differences were analyzed with the Statistica^TM^ software using two-way analysis of variance (ANOVA) and the least significant difference (LSD) post hoc test. For analysis of data obtained with the same animals evaluated on several occasions (e.g., time-course experiments), between–within univariate or multivariate ANOVA for repeated measures was used (with time being the repeated measure). Data from paired-pulse electrophysiology experiments and *c-fos* mRNA distribution histogram were analyzed by between–within univariate or multivariate ANOVA for repeated measures (with interpulse interval and number of grain/cell being the repeated measures, respectively).

## Results and discussion

### Direct interactions of the D_3_ receptor with glutamate

In rat brain, the largest receptor densities occur in granule cells of the islands of Calleja and in medium-sized spiny neurons of the rostral and ventromedial shells of nucleus accumbens, which coexpress the D_1_ receptor, substance P, dynorphin, and/or neurotensin (Diaz et al. 1995; Le Moine and Bloch 1996). These output neurons from the nucleus accumbens receive their dopaminergic innervations from the ventral tegmental area and reach the entorhinal and prefrontal cortex after relays in the ventral pallidum and mediodorsal thalamus. In turn, the shell of nucleus accumbens receives projections from the cerebral cortex (infralimbic, ventral, agranular, insular, and piriform areas), hippocampus, and amygdala and also projects to the ventral tegmental area from which dopaminergic afferents originate (Zahm and Brog 1992; Pennartz et al. 1994). These various specific connections of the shell of nucleus accumbens, a part of the “extended amygdala” (Heimer 2000), suggest that this area is involved in a series of feedback or feed-forward loops, involving notably the prefrontal cortex and ventral tegmental area and subserving control of emotions, motivation, and reward. In the human and non-human primate brains, the phenotype of neurons expressing the D_3_ receptor is not yet identified, but several studies show their distribution to be rather similar to that in the rat, with, however, higher densities and larger distribution in the ventral part of the caudate putamen and the cerebral cortex (Landwehrmeyer et al. 1993; Hall et al. 1996; Girgis et al. 2011; Gallezot et al. 2012).

**Fig. 1:**
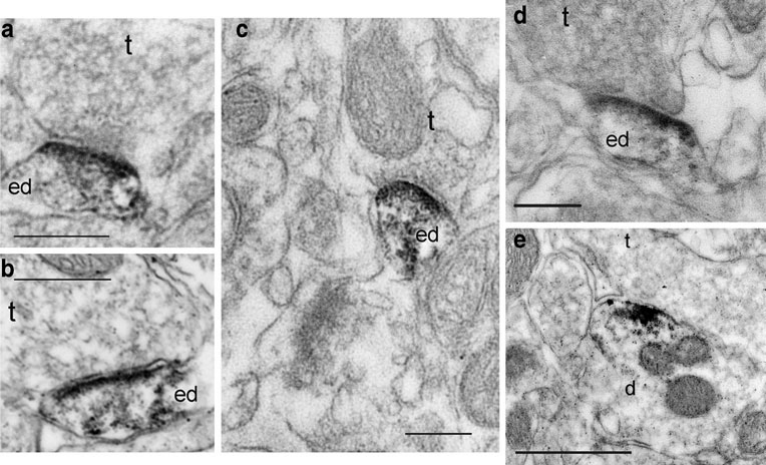
Immunoperoxidase labeling for the D_3_ receptor in the nucleus accumbens of rat brain. Electron micrographs of the nucleus accumbens shell immunostained by the avidin–biotin–peroxidase complex (ABC) method to reveal immunoreactivity for the D_3_ receptor (anti-D_3_ receptor antibody, 1: 2,000; see Diaz 2000). Images (**a–d**) showing that immunoreactivity for D_3_R, is localized at asymmetric synapses in dendritic spines (*ed*) in medium-sized spiny neurons of the nucleus accumbens. Note the high density of labeling in, or near, the region of the post-synaptic density at the head of dendritic spines (particularly visible in a sagittal section of a dendritic spine in **d**), which is juxtaposed to presynaptic terminal profiles (*t*), containing clear vesicles and forming asymmetric synapses. Image (**e**) shows immunoreactivity for D_3_R in a dendrite profile (*d*) apposed to an unlabeled axon terminal (*t*) that forms a symmetric synapse. *Scale bar* 0.25 μm

**Fig. 2:**
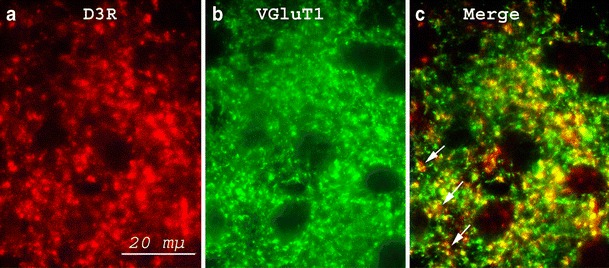
Colocalization of D_3_ receptor (**a**) and VGluT1 (**b**) immunoreactivity in the nucleus accumbens. Representative distribution of fluorescent signals generated in double immunostained rat brain sections using a rabbit polyclonal antibody directed against the D_3_ receptor (D_3_R) and guinea pig polyclonal antibodies directed against the vesicular glutamate transporter 1 (VGluT1) to reveal association of post-synaptic D_3_R with presynaptic glutamatergic inputs. **a** Dot-like red-fluorescent immunoreactivity displayed for the D_3_R. **b** Dense immunofluorescent staining for the VGluT1. **c** Close association of a large-proportion D_3_R immunostaining with presynaptic glutamatergic terminals immunolabeled with the anti-VGluT1 antibody

We carried out ultrastructural analysis to determine the subcellular distribution of the D_3_ receptor in the nucleus accumbens of rats. We have used electron transmission microscopy and immunostaining with a specific anti-D_3_ receptor antibody, which was thoroughly validated by immunoprecipitation of solubilized D_3_ receptor binding, overlapping of immunolabeling with D_3_ receptor binding, and suppression of immunolabeling in D_3_ receptor-deficient mice (Diaz et al. 2000). The peroxidase immunoreactivity was often detected at the level of dendritic spines in medium-sized spiny neurons. Figure 1a–d shows four examples of electron-dense peroxidase reaction product concentrated at the level of asymmetric synapses at the head of dendritic spines. We estimated that 64 % of immunolabeling was associated with asymmetric synapses (30 positive over 47). The synaptic localization of the D_3_ receptor is in marked contrast with those of D_1_ and D_2_ receptors, which are either perisynaptic or spread all over dendrites and dendritic spines in medium-sized spiny neurons of striatum (Hersch et al. 1995; Delle Donne et al. 1997) and nucleus accumbens (Hara and Pickel 2005; 2007; Pickel et al. 2006). Very surprisingly, most of the D_3_ receptor-positive synapses are of asymmetric type, which is a typical feature of glutamatergic synapses (Uchizono 1965; Kemp and Powell 1971), whereas the majority of presumed dopamine terminals form symmetric synapses (Bolam et al. 2000; Arluison et al. 1984). Immunoreactivity associated to symmetric synapses (Fig. 1) was rarely observed in this study. Interestingly, in the examples shown, labeled synapses are all contained in the head of spines, particularly identifiable in the sagittal section of a spine depicted in Fig. 1d. Cortical glutamatergic terminals form asymmetric synapse at the head of dendritic spines on medium-sized spiny neurons of striatum, whereas other afferences mainly contact dendritic necks or shafts (Bolam et al. 2000). The localization of D_3_ receptor labeling at the vicinity of glutamatergic terminals was confirmed by double fluorescence labeling experiments (Fig. 2) with an antibody directed against the vesicular transporter 1 (VGluT1), a marker of glutamatergic terminals of neurons originating from the cerebral cortex, amygdala, or hippocampus, but not thalamus, which express VGluT2 (Hartig et al. 2003). Figure 2 shows that VGluT1 and D_3_ receptor immunofluorescences are detected as tiny puncta occupying the neuropil and displaying a clear association. Of course, the resolution of the technique does not permit us to go deep into the physical association between the two immunomarkers. Nevertheless, there seems to be no strict colocalization of D_3_ receptor and VGluT1; rather, they appear apposed each other, particularly in the left bottom part of Fig. 2 (*arrows*), possibly reflecting the glutamatergic terminal/dendritic spine apposition. Thus, both ultrastructural study and double immunofluorescence experiments suggest the presence of the D_3_ receptor at glutamatergic synapses in medium-sized spiny neurons of nucleus accumbens.

There are two implications to the peculiar localization of the D_3_ receptor at glutamatergic synapses. The first one deals with a fundamental issue of neurotransmission that is volume transmission. Classical fast neurotransmission by excitatory (glutamate, aspartate) or inhibitory (γ-amino-butyric-acid (GABA), glycine) amino acids operates through voltage- and calcium-dependent release of the neurotransmitter into the synaptic cleft, stimulation of neurotransmitter receptors located at the other edge of the synaptic cleft in specialized post-synaptic domains, and termination of the signal by receptor desensitization and neurotransmitter reuptake. Other neurotransmitters, such as monoamines or neuropeptides, can exert their action through a second, much slower, non-synaptic diffuse mode of neurotransmission called volume transmission (Zoli et al. 1999). Considering that asymmetric synapses bearing the D_3_ receptor are formed with edges of glutamatergic terminals located at some distance of dopamine terminals, the D_3_ receptor transmission uses a peculiar and unprecedented variety of volume transmission, in which released dopamine diffuses and dilutes over some distance in the interstitial space, to stimulate post-synaptic receptors of distal non-dopaminergic synapses. This mode of transmission also differs from “spillover” transmission of glutamate or GABA, in which the released neurotransmitter diffuses before targeting a distal homologous synapse (Kullmann 2000). Volume transmission through the D_3_ receptor is possible owing to its higher affinity for dopamine, as compared to other dopamine receptor subtypes (Sokoloff et al. 1990).

The second issue is the functional role of D_3_ receptor at glutamatergic synapses, which bear α-amino-3-hydroxy-5-methyl-4-isoxazole-propionic acid (AMPA) and *N*-methyl-d-aspartate (NMDA) receptors (Bernard et al. 1997; Bernard and Bolam 1998) at the post-synaptic site. Liu and colleagues elegantly addressed this question (Liu et al. 2009). They showed that the D_3_ receptor binds to Ca^2+^/calmodulin-dependent protein kinase II (CaMKII) in a Ca^2+^-dependent manner. This interaction involves the third intracellular loop of the receptor and occurs in the post-synaptic densities (PSD)-enriched fraction of accumbal neurons, but not in other D_3_-expressing neurons in the substantia nigra or ventral tegmental area. PSD are associated with glutamatergic synapses, which confirms the site of interactions. NMDA receptor activation that increases intracellular Ca^2+^ stimulates D_3_ receptor–CaMKII interactions. D_3_ receptor–CaMKII interactions further impact downstream effectors of cyclic AMP/protein kinase A, such as other constituents of the glutamatergic synapses, such as AMPA receptor GluR1 subunit. In turn, D_3_ receptor–CaMKII interactions downregulate D_3_ receptor functions. A reciprocal regulation of glutamate and dopamine at synaptic glutamatergic synapses is a result from this process, by which D_3_ receptor function is exacerbated in conditions of NMDA receptor blockade, a situation that mimic psychotic symptoms. Indeed, phencyclidine (PCP), a non-competitive antagonist at the NMDA receptor, is an anesthetic agent that has dissociative and psychotomimetic properties in man (Javitt and Zukin 1991). PCP produces a state of sensory isolation, seeming “drunkenness” and hallucinations, often followed by a feeling of depersonalization in abusers (Snyder et al. 1981), and schizophrenic-like symptoms in healthy volunteers (Jentsch and Roth 1999; Luby et al. 1959). Psychotic symptoms induced by PCP, or the pharmacologically related compound ketamine, include both positive (hallucinations, delusions) and negative (formal thought disorder, social withdrawal) symptoms, as well as cognitive dysfunction (Cosgrove and Newell 1991). Therefore, the considerations above support the view that blockade of D_3_ receptor could reverse the effects of NMDA receptor blockade and elicit antipsychotic-like actions.

### Indirect interactions of the D_3_ receptor with glutamate

The glutamate theory owned its birth from the fortuitous observation that PCP and ketamine, originally developed as anesthetics, possessed psychotomimetic potential. Eventually, they were discovered to be non-competitive NMDA receptor blockers (Javitt and Zukin 1991). Subsequently, a theory was developed postulating a reduced NMDA function in schizophrenia (Goff and Coyle 2001). However, the concept has progressively emerged that the dysfunction of glutamate was rather hyperfunction, through exacerbated glutamate release from hyperexcitable cortical pyramidal cells and activation of AMPA receptors. The most conceptualized version of the glutamate theory (Moghaddam and Javitt 2012) now indicates that hyperexcitability of pyramidal cells results from a loss of inhibition of GABAergic interneurons, which can be mimicked by blocking NMDA receptors expressed by these neurons. This results in enhanced electrical spike activity and disorganization of the firing of cortical neurons, adding “noise” and interrupting the ability of these neurons to process information. This also results in enhanced glutamate by pyramidal cells that project to various subcortical areas, including striatum and nucleus accumbens.

Anatomically, dopamine-immunoreactive terminals of the mesocortical pathway converge on pyramidal cells and parvalbumin-positive fast-spiking interneurons (Gaspar et al. 1995; Verney et al. 1990; Sesack et al. 1995; 1998; Gorelova et al. 2002; Sesack et al. 1995; 1998). The activity of cortical cells is controlled by dopamine in a rather complex manner because D_1_ and D_2_ receptors can exert both facilitation or inhibition of cell firing on excitatory pyramidal cells and the inhibitory interneurons (Seamans and Yang 2004). Schematically, D_1_ receptor-mediated effects include an increase in NMDA currents, a decrease in glutamate release, and an increase in interneuron excitability and inhibitory post-synaptic current amplitude in pyramidal cells, whereas D_2_ receptor-mediated effects include a reduction of inhibitory post-synaptic currents onto pyramidal cells and reduction of NMDA currents and spiking. This complex modulation by dopamine of the prefrontal cortex maintains coherent activity that is crucial for task-dependent neuronal activity, decision-making assessed with delay discounting and effort-based procedures (Goldman-Rakic et al. 2004; Seamans and Yang 2004; Floresco and Magyar 2006).

D_3_ receptors are virtually absent from the prefrontal cortex (Bouthenet et al. 1991; Diaz et al. 1995), but local injections of selective D_3_ antagonists in the prefrontal cortex induced cognitive improvement (Loiseau and Millan 2009), suggesting that, despite their low expression in this brain area, the functional role of D_3_ may not be negligible. In medium-sized GABAergic neurons of the nucleus accumbens, the D_3_ receptor is not only expressed on the dendritic spines, but also on terminals of these neurons projecting to the lateral part of the ventral tegmental area and medial part of substantia nigra of the mesencephalon. This was demonstrated by lesions with kainate of the accumbal neurons, which produced a similar decrease in D_3_ receptor binding at the site of the lesion and in the mesencephalon (Diaz et al. 2000). The expression of the D_3_ receptor at the terminals of descending accumbal GABAergic neurons makes the major part of the D_3_ receptor binding in the ventral tegmental area/substantia nigra, which can be labeled as the selective D_3_ receptor ligands [^3^ H]-7-OH-DPAT (Levesque et al. 1992) or [^125^I]-7-OH-PIPAT (Stanwood et al. 2000) in receptor autoradiography experiments on brain slices, as well as in PET studies with [^11^C]-(+) PHNO (Narendran et al. 2006; Graff-Guerrero et al. 2009b; Rabiner et al. 2009; Gallezot et al. 2012). In addition, D_3_ receptors are also expressed by dopamine neurons in the ventral tegmental area and substantia nigra: D_3_ receptor mRNA is expressed in dopamine neurons (Diaz et al. 1995), and its expression decreases after lesion of these neurons by 6-hydroxydopamine (Lévesque et al. 1995); all dopamine neurons identified by positive immunoreactivity to tyrosine hydroxylase also express the D_3_ receptor protein detected by immunoreactivity (Diaz et al. 2000).

It can be hypothesized that the D_3_ receptor exerts a tonic inhibition on dopamine neurons in the ventral tegmental area projecting to the nucleus accumbens, either by stimulating GABA release at accumbal neuron terminals or by an autoreceptor control. Several lines of evidence support this conclusion. First, dopamine release (Tang et al. 1994) and synthesis (O'Hara et al. 1996) are inhibited by stimulation of the D_3_ receptor expressed in a transfected mesencephalic cell line and various agonists, with limited preference for the D_3_ receptor (Sautel et al. 1995), and inhibit dopamine release and synthesis and neuron electrical activity (Levant 1997), giving support to the existence of D_3_ autoreceptors. However, the selectivity of these agonists toward the D_3_ receptor in vivo has been strongly questioned because they elicit similar inhibition of dopamine neuron activities in wild-type and D_3_ receptor-deficient mice (Koeltzow et al. 1998). In addition, dopamine autoreceptor functions are suppressed in D_2_ receptor-deficient mice (Mercuri et al. 1997; L'hirondel et al. 1998). Nevertheless, dopamine extracellular levels in the nucleus accumbens (Koeltzow et al. 1998) and striatum (Joseph et al. 2002) are twice as high in D_3_ receptor-deficient as in wild-type mice, suggesting a control of dopamine neurons activity by the D_3_ receptor. The hypothesis of a control exerted by D_3_ autoreceptors is also supported by the observations that D_3_ receptor-deficient mice display signs reminiscent of hyperdopaminergia, presumably resulting from the lack of autoreceptors controlling dopamine neuron activity (Accili et al. 1996). Furthermore, blocking the D_3_ receptor by selective antagonists increases extracellular levels of dopamine in the prefrontal cortex (Lacroix et al. 2003), a projecting area of mesencephalic dopamine neurons.

Hence, the negative control that the D_3_ receptor may exert on dopamine neurons, directly by its autoreceptor function or indirectly through the control of GABA release, results in a downregulation of dopamine release in the prefrontal cortex and, consequently, an excitation of glutamate pyramidal cells. This control is relevant to schizophrenia because it is now well accepted that dopamine deficiency in the prefrontal cortex is a hallmark of the disease (Davis et al. 1991; Abi-Dargham 2004; Carlsson and Carlsson 2006). This feature has recently received a strong support from a PET studies, showing that amphetamine-induced dopamine was *reduced* in the prefrontal cortex of schizophrenic patients (Abi-Dargham 2011), although it has been repeatedly shown to be *increased* in subcortical areas such as the striatum (Laruelle et al. 1996; Abi-Dargham et al. 1998). Furthermore, D_3_ receptor expression may be upregulated in the post-mortem brain of schizophrenic patients (Gurevich et al. 1997), which may contribute to accentuating dopamine deficiency in the prefrontal cortex; however, upregulation of D_3_ receptors in schizophrenia has not been confirmed in one PET study (Graff-Guerrero et al. 2009b). It is therefore conceivable that D_3_ receptor antagonists, by relieving the break exerted by D_3_ receptors onto dopamine mesocortical neurons, may correct dopamine deficiency in the prefrontal cortex and normalize glutamate pyramidal cell activity.

**Fig. 3:**
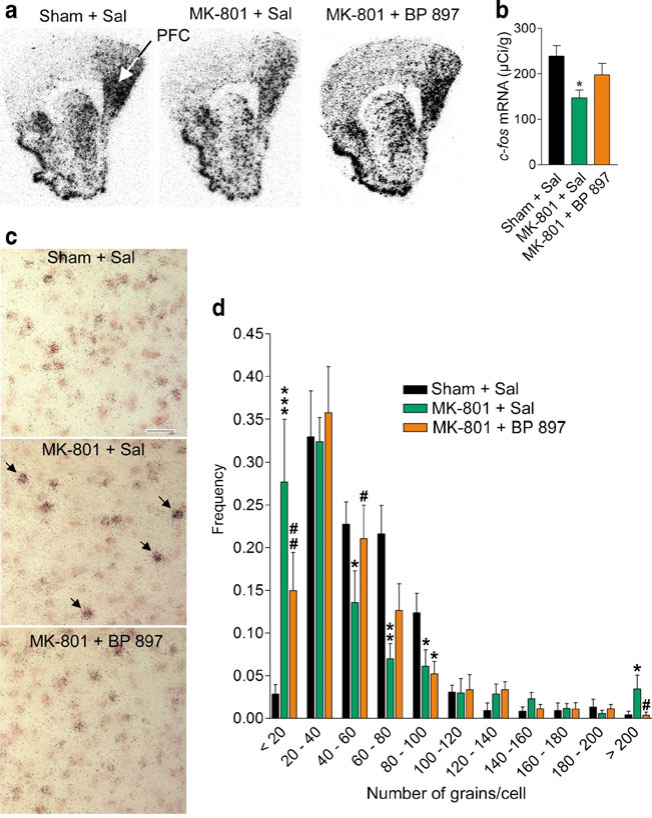
Functional imaging measured by *c-fos* expression in mPFC after chronic NMDA-R blockade by MK-801, showing mPFC dysfunction reversed by BP 897. Animals received a 7-day continuous infusion of saline and a saline subchronic treatment (Sham + Sal) or a continuous infusion of MK-801 and a subchronic 4.5-day treatment with either saline (MK-801 + Sal) or BP 897 (1 mg kg^-1^, MK-801 + BP 897), as described in “Methods.” Then, at day 10, they were sacrificed, and the brain was processed for double in situ hybridization with a [^33^P]-labeled *c-fos* cRNA probe and a digoxigenin-labeled cRNA probe that corresponds to the sequence encoding the *vesicular glutamate transporter type I* (*VGluT1*), a marker of glutamatergic neurons. **a** Film autoradiography of *c-fos* mRNA signals. Coordinate from bregma 1.94 mm. **b** Quantitative analysis of autoradiographic pictures of *c-fos* mRNA signals in the mPFC. Results are mean ± SEM. (*n* = 5–6). Significant effect of treatment (*F*_2,14_ = 4.24, *P* = 0.036), *P < 0.02 vs. Sham + Sal or MK-801 + BP 897. **c** Microphotographs showing *c-fos* (*dark dots*) and *VGluT1* (*pink color*) signals. Treatment with MK-801 induced the appearance of heavily labeled neurons (*arrows*). *Bar*: 50 μm. **d** Distribution of *c-fos*-labeled neurons according to *c-fos* mRNA level (measured as number of grain per cell) in the prelimbic cortex subregion of the mPFC. On each animal, 60 to 80 neurons were analyzed, and there were five to six animals per treatment group. Significant treatment × distribution interaction (*F*_20,150_ = 3.38, *P* < 0.0001). *P < 0.05; **P < 0.01 vs. Sham + Sal; ***P < 0.001; ^#^ *P* < 0.05; ^##^ *P* < 0.01 vs. MK-801 + Sal

The normalization of glutamate activity by D_3_ receptor blockade was studied using functional neuroimaging based on quantification of *c-fos* mRNA expression, a non-specific marker of neuronal activity, in a mouse model of chronic NMDA receptor blockade. Indeed, chronic exposure of humans to NMDA antagonists seems to mimic schizophrenia better than acute exposure: Hallucinations are mainly auditory and are accompanied by negative symptoms and cognitive deficits (Jentsch and Roth 1999). Chronic NMDA receptor blockade was obtained by a 7-day continuous infusion of the highly potent and selective non-competitive NMDA antagonist dizocilpine (MK-801). MK-801 was infused (0.02 mg kg^-1^ h^-1^) via subcutaneously implanted osmotic minipumps from day 1 to 7. To specifically block the D_3_ receptor, we used BP 897, a D_3_ receptor-selective partial agonist with an intrinsic activity of ~0.5 in a heterologous expression system (Pilla et al. 1999), which also has antagonist properties in vivo (Preti 2000). BP 897 was subchronically administered for 4.5 days at a dose of 1 mg kg^-1^ i.p. twice a day, starting 4.5 days after minipump implantation (day 5 to 9). The animals were sacrificed at day 10, when MK-801 and BP 897 no longer have acute effect but when some behavioral abnormalities were still present in MK-801-treated mice and normalized by BP 897 subchronic treatment (unpublished results). When observed and quantified on film autoradiographs, *c-fos* mRNA levels were significantly reduced in the medial prefrontal cortex (mPFC) after treatment with MK-801, and this effect was at least reduced after BP 897 administration (Fig. 3a, b). No trend toward changes in *c-fos* expression was observed in other brain regions (not shown). The effects of BP 897 alone were not quantified in these experiments, but a pharmacological analog of BP 897 did not increase *c-fos* expression in the prefrontal cortex (Southam et al. 2007). We performed a more precise analysis and grain counting at the microscopic level after double hybridization with a cRNA probe that labels transcripts of *VGluT1*. At the level of mPFC in control animals, 54 ± 2 % (*n* = 6, 630–828 neurons counted per animal) of *VGluT1*-positive neurons expressed *c-fos* (Fig. 3c), and this percentage did not change after treatment (56 ± 3 %, *n* = 6, and 54 ± 3 %, *n* = 6, in animals receiving MK-801 and MK-801 + BP 897, respectively). The number of *c-fos*-positive neurons exceeded by 25–40 % the number of double-positive neurons, indicating that *c-fos* is mainly, but not exclusively, an index of glutamatergic neuron activity in the mPFC. Analysis of the distribution of *c-fos*-positive neurons revealed that *c-fos* mRNA levels globally decreased following treatment with MK-801 in a large fraction of the neuronal population, expressing moderate to low levels of *c-fos* mRNA (number of grains lower than 20 or comprised between 40 and 60), which is shown by the leftward shift of the distribution curve (Fig. 3d). The distribution curve after MK-801 + BP 897 was more similar to that obtained in control animals. In animals receiving MK-801, we also observed the appearance of heavily *c-fos*-labeled neurons (>200 grains/cell, arrows in Fig. 3c), which were all VGluT1 positive and rarely encountered in the other experimental conditions. The results indicate that NMDA receptor blockade induced changes in mPFC function, including both neuronal hypofunction and heterogeneous hyperactivity.

**Fig. 4:**
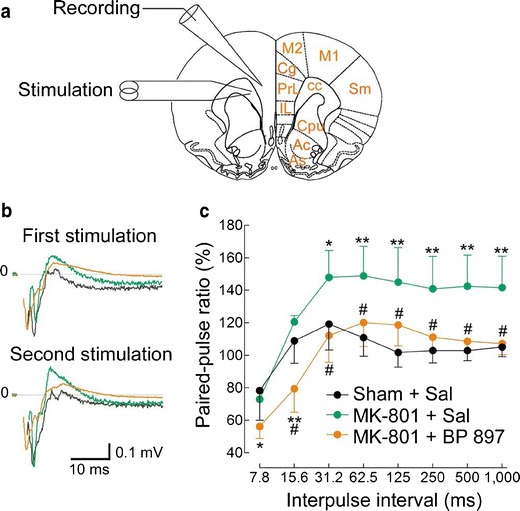
Neuronal hyperresponsiveness in prefrontal cortex after chronic NMDA-R blockade by MK-801, assessed by paired-pulse facilitation, and normalization by BP 897. Slices were prepared from animals receiving continuous infusion of saline and a saline subchronic treatment (Sham + Sal) or a continuous infusion of MK-801 and a subchronic treatment with either saline (MK-801 + Sal) or BP 897 (1 mg kg^-1^, MK-801 + BP 897) sacrificed 3 days after continuous MK-801 cessation and at least 16 h after the last injection. **a** Field potentials were recorded with an electrode placed in the middle layers of the prelimbic cortex (PrL) after stimulation in the adjacent region. *Ac* nucleus accumbens core, *As* nucleus accumbens shell, *cc* corpus callosum, *Cg* cingulate cortex, *Cpu* caudate-putamen, *IL* infralimbic cortex, *M1* primary motor cortex, *M2* secondary motor cortex, *Sm* primary sensorimotor cortex. **b** Field potentials after paired-pulse stimulation with an interpulse interval of 62.5 ms. Traces are means of eight successive recordings performed on a representative animal of each group treatment. Artifacts immediately following stimulation were removed for clarity. Color codes as in **c**. **c** Paired-pulse facilitation, measured as the ratio of amplitudes of responses to the second and first stimulations, as a function of the interpulse interval. Results are mean ± SEM. of averaged ratios recorded in five to six animals. There were significant effects of interpulse interval (*F*_7,98_ = 16.55, *P* < 0.00001). *P < 0.05; ** *P* < 0.01

In the same animal model, i.e., continuous 7-day MK-801 treatment and 4-day subchronic BP 897 treatment, the changes in the electrical activity of mPFC efferent glutamatergic neurons were assessed by measuring paired-pulse facilitation of field potentials after orthodromic stimulation in slices. For this, a recording electrode was placed in the middle layer of the prelimbic cortex (PrL), a part of mPFC containing glutamatergic neurons that provide direct excitatory inputs to the shell part of nucleus accumbens, where the D_3_ receptor is expressed (Fig. 4a). Trains of double pulses, with varying interpulse intervals, were applied on afferent excitatory fibers to the mPFC with a bipolar electrode placed on the underlying white matter (Fig. 4a). At a short interpulse interval (7.8 ms) in control animals, paired-pulse depression was observed, i.e., the amplitude of the response to the second pulse was lower than that to the first (Fig. 4c). At an interpulse interval of 31.2 ms, paired-pulse facilitation (~ +20 %) was observed and disappeared at longer intervals (>62.5 ms). In animals receiving MK-801, paired-pulse facilitation was largely and significantly increased (> + 40 % at an interval of 62.5 ms; see Fig. 4b) and was maintained elevated at long intervals (up to 1,000 ms; see Fig. 4c). In animals receiving MK-801 + BP 897, paired-pulse depression was significantly increased for short interpulse intervals (<31.2 ms) as compared to control animals, and paired-pulse facilitation returned to control at longer intervals (Fig. 4b, c). These results demonstrate long-lasting hyperresponsiveness of mPFC efferent glutamatergic neurons after NMDA hypofunction and normalization by D_3_ receptor blockade. This effect is supposed to be exerted indirectly, but further studies will be necessary to address whether it involves modulation of dopamine release in the prefrontal cortex.

### D_3_ receptor antagonists in preclinical models of schizophrenia based on *N*-methyl-d-aspartate receptor blockade

The psychotic effects of dissociative anesthetics acting by blocking the NMDA receptor in humans (see above) represent not only a conceptual framework for understanding schizophrenia, but also an impressive pharmacological model of the disease. Indeed, behavioral abnormalities elicited by PCP, ketamine, or MK-801, a more selective NMDA receptor blocker, have been used to model symptoms of schizophrenia in animals and to detect antipsychotic-like properties of drugs (Corbett et al. 1995; Jentsch and Roth 1999; Bradford et al. 2010; Adell et al. 2012). In rodent, these drugs elicit hyperactivity and stereotypies that can be reversed by treatment with antipsychotic drugs. We have shown that hyperactivity induced by MK-801 was largely reduced in D_3_ receptor-deficient mice and readily antagonized by the D_3_ receptor-selective partial agonist BP 897 (Leriche et al. 2003). We have now extended this observation to several D_3_ receptor-selective antagonists (Table 1). In the test, we are measuring the effects of tested agents on both spontaneous locomotor activity and MK-801-induced hyperactivity (Leriche et al. 2003). In general, D_3_ receptor-selective antagonists or partial agonists reduced MK-801-induced hyperactivity at lower doses than those that reduced spontaneous activity. This was not the case of haloperidol ((Leriche et al. 2003) Table 1).

**Table 1:** Effects of D_3_ receptor antagonists and partial agonists, compared to selected antipsychotics in the MK-801 test in the mouse

Compound	D_3_/D_2L_ selectivity^a^	ED_50_ for inhibiting spontaneous activity^b^ (mg kg^-1^ i.p.)	ED_50_ for inhibiting MK-801-induced hyperactivity^c^ (mg kg^-1^ i.p.)	Ratio ED_50_ (spontaneous/MK-801 induced)
Aripiprazole	0.32	0.48	0.21	2.3
Haloperidol	0.41	0.21	0.08	2.6
Clozapine	0.52	6.3	0.49	13
Cariprazine	5.8	0.11	0.02	5.5
ABT-925	17.3	9.8	< 0.16	>61
BP 897	281	7.7	0.40	19
S33084	324	>10	1.5	6.6
SB-277011A	871	28	14	2
F17141	151	>10	0.28	>36

^a^Ratio of *K*_i_ values for inhibiting [^3^ H]-spiperone binding at recombinant human D_2L_ and D_3_ receptors (Newman-Tancredi et al. 2007)

^b^Spontaneous locomotor activity was recorded during the 30-min phase of habituation in the actimetry cage

^c^Hyperactivity was measured during 90 min following injection of MK-801 (0.14 mg kg^-1^ i.p.)

**Fig. 5:**
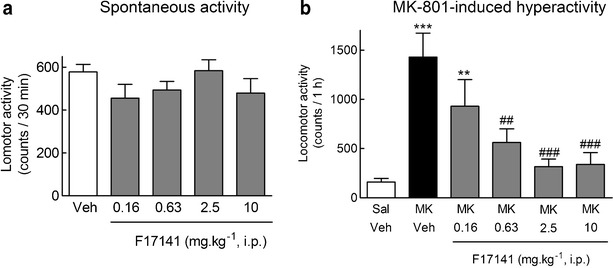
F17141 had no effect on spontaneous horizontal locomotor until 10 mg kg^-1^ i.p. (**a**) and dose dependently antagonized the stimulant effects of acute NMDA-R blockade by MK-801 (**b**) in mice. F17141 (0.16–10 mg kg^-1^, i.p.) or saline (Sal) was injected, and spontaneous behaviors were recorded during 30 min (**a**). MK-801 (0.14 mg kg^-1^, i.p., MK) or Sal was then administered, and behavior was recorded for a subsequent 1-h period (**b**). Results are mean ± SEM. of *N* = 10–20 animals (**a**) and *N* = 10 animals (**b**). **P < 0.01; ***P < 0.001 vs. Sal (**a**) or Sal + Sal (**b**) and ^##^ *P* < 0.01 and ^###^ *P* < 0.005 vs. MK + Sal (**b**), by ANOVA followed by LSD post hoc test. ANOVA showed no overall effect of F17141 on spontaneous activity (*F*_4,55_ = 1.4, *P* = 0.23), but a significant effect on MK-801-induced hyperactivity (*F*_5,53_ = 5.6, *P* = 0.00035)

We also tested F17141, a novel D_3_ antagonist, which exhibits a 355 time higher affinity at D_3_ receptors compared to D_2_ receptors (Sokoloff et al. 2008). Interestingly, F17141 completely antagonized the MK-801 stimulant effect and did so with little or no effect on spontaneous activity (Fig. 5), suggesting an antipsychotic-like effect at doses much lower than purely sedative ones. In this respect, the profile of F17141 is more similar to that of clozapine and of other D_3_ receptor antagonists than that of haloperidol ((Leriche et al. 2003) (Table 1).

**Fig. 6:**
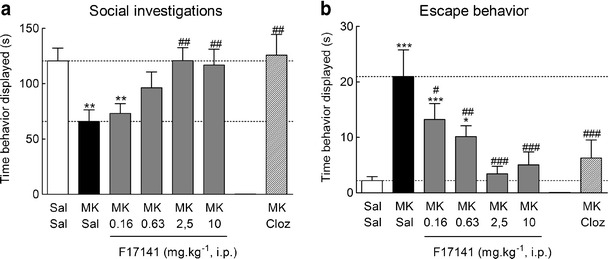
Reversion by F17141 and clozapine of social interaction deficits induced by chronic NMDA-R blockade by MK-801 in mice. A continuous 7-day treatment with MK-801 (0.02 mg kg^-1^ h^-1^, s.c.) induced a robust reduction of social interaction time (**a**, *P* = 0.0014, by LSD post hoc test) and an increase in escape behavior (**b**, *P* = 9.7 × 10^-8^, by LSD post hoc test) displayed by the resident mice compared to saline controls. (**a**) F17141 (0.16–10 mg kg^-1^ i.p.) acutely administered 30 min before the test dose dependently, and completely at the highest doses, reversed the social interaction time deficits (**a**, ANOVA *F*_5,57_ = 4.0, *P* = 0.0033), and the escape behavior time increase (**b**, ANOVA *F*_5,57_ = 9.3, *P* = 1.6 × 10^-6^). Clozapine (*Cloz*), used as an internal control, administered acutely at the challenge dose of 1 mg kg^-1^ i.p. 30 min before the test, completely reversed social investigation deficits (**a**, ANOVA *F*_2,38_ = 8.85, *P* = 6.9 × 10^-4^) and the exacerbated escape behavior induced by MK-801 (**b**, ANOVA *F*_2,38_ = 15.9, *P* = 9.3 × 10^-9^). Results are mean ± SEM of *N* = 8–15 animals. *P < 0.05, **P < 0.01, and ***P < 0.005 vs. Sal + Sal and ^#^ *P* < 0.05, ^##^ *P* < 0.01, and ^###^ *P* < 0.005 vs. MK + Sal, by ANOVA followed by Fischer’s LSD post hoc test

Seven-day continuous infusion of MK-801 (0.02 mg kg^-1^ h^-1^) via subcutaneously implanted osmotic minipumps elicited social interaction deficits in mice in a “resident–intruder” paradigm adapted from (Dixon et al. 1994; Mohn et al. 1999) with some modifications. In the original test, a resident mouse is housed alone for 2 weeks, and then, a group-housed intruder is introduced to the resident cage. In our experiments, for convenience of observation, group-housed intruder and isolated resident were not confronted in the resident cage but in a test arena, to which both where individually habituated before confrontation. Even with that modification, the behaviors displayed by both groups of mice were almost the same as those observed in the original conditions cited above (Leriche, personal observations). Typically, the resident actively initiates social investigations of the intruder, sometimes initiating fights, and rarely avoids social interaction by escape (Dixon et al. 1994; Mohn et al. 1999). Social interaction deficits in the resident–intruder test consisted of both a reduction in social investigations and an increase in escape behavior displayed by the resident mice. Acute treatment with F17141 of the resident and intruder, 30 min before confrontation, dose dependently and completely inhibited the social investigation deficit and escape behavior induced by MK-801, with ID_50_ values of 0.49 and 0.32 mg kg^-1^ i.p., respectively (Fig. 6). Clozapine, at a dose of 1 mg kg^-1^ i.p., also completely inhibited the social interaction deficits and escape behavior in this same test (Fig. 6), as well as did BP 897, at a dose of 1 mg kg^-1^ i.p. (not shown). However, haloperidol was inactive at 0.1 mg kg^-1^, a dose at which it does not produce catalepsy (not shown).

Taken together, the results obtained with F17141 and other D_3_ receptor antagonists indicate that blocking the D_3_ receptor produces antipsychotic-like effects in mouse models mimicking both positive and negative symptoms. These results are consistent with the observations that disruption by NMDA receptor blockers of glutamate homeostasis leads to hyperexcitation of pyramidal cells and increased glutamate release in the prefrontal cortex (Moghaddam and Javitt 2012) and presumably subcortical areas including the nucleus accumbens and with the evidence presented above that D_3_ receptor antagonists can oppose the effects of NMDA blockade by acting directly at post-synaptic levels and indirectly at presynaptic levels on pyramidal cells. Moreover, D_3_ antagonists have consistently showed ability to increase cognitive performance or to reverse cognitive deficits in rodents and monkeys (Sigala et al. 1997; Loiseau and Millan 2009; Laszy et al. 2005; Millan et al. 2010; Watson et al. 2012a; Watson et al. 2012b). They also support the use of D_3_ receptor-selective antagonists in the treatment of positive and negative symptoms of schizophrenia, as well as cognitive deficits associated with the disease.

### Clinical studies of D_3_ antagonists in schizophrenia

To date, two D_3_ receptor-selective compounds have reached clinical proof-of-concept studies in schizophrenia, and the results were reported. BP 897 was the first to be clinically assessed in 2003 in patients with schizophrenia (Lecrubier 2003). It was a 4-week double-blind randomized placebo-controlled study, enrolling 77 patients with schizophrenia. Population sample was hospitalized schizophrenics with a paranoid, disorganized, or undifferentiated subtype according to DSM IV, presenting an acute episode of exacerbation; 52 patients were randomized to BP 897 (10 mg b.i.d.) and 25 to placebo. Plasma was collected at 8 and 28 days to assay BP 897. A minimal score on positive symptoms (PANSS subscore > 15) and on negative symptoms (PANSS subscore > 15) was required as well as a CGI > 4. After a 3–7-day placebo wash-out period, those with less than 30 % improvement could be included. The primary efficacy criterion was the difference between the PANSS total score at baseline and after 4 weeks of treatment. Early dropouts were 40 % in the BP897 group and 56 % in the placebo group, mainly related to the absence of efficacy in both groups; no adverse event emerged as frequent and/or responsible for early dropout. In the intention-to-treat population, no difference was observed between BP 897 and placebo on the primary efficacy criterion. In the per protocol population treated for at least for 1 week, the mean difference of change between BP 897 (PANSS initial: 84.2 ± 14.9, final: 68.3 ± 22, *n* = 44) and placebo (PANSS initial: 84 ± 15.4, final: 73.6 ± 18.8, *n* = 17) was 10.75 (*p* = 0.024) when adjusting for sex, center, and study duration. Plasma levels were available in 33 patients treated with BP 897 and were compared to the active BP897 plasma levels in rodent and monkey models (~200 nM; see (Bezard et al. 2003): 11 patients had low BP 897 plasma levels (<50 nM at day 8 or 28), and their mean improvement on the PANSS was 7.1 versus 22.9 in the 22 other patients. Patients with the highest plasma levels (>100 nM at days 8 and 28) show the best improvement with a mean decrease from baseline of 26.9. No relevant adverse event was identified specially at the extrapyramidal level. Although the efficacy of BP 897 in acute or subacute schizophrenic patients was not demonstrated in this study, probably related to a low dose not reaching therapeutic levels, there was a strong trend toward an antipsychotic effect dependent on plasma exposure, supporting the hypothesis that a D_3_ receptor antagonist or partial agonist could exhibit antipsychotic properties.

The second proof-of-concept clinical study assessed the efficacy of ABT-925 and can be summarized as follows. In a 6-week, randomized, placebo controlled clinical study (Redden et al. 2011), ABT-925 at doses of 50 and 150 mg per day failed to produce antipsychotic effects, as measured on the PANSS total score. However, a subsequent PET study with [^11^C]-(+)-PHNO in healthy volunteers showed that, at the doses used in the clinical study in patients suffering from schizophrenia, probably less than 40 % of cerebral D_3_ receptors were occupied by ABT-925 (Graff-Guerrero et al. 2010), suggesting that the doses used in the clinical study were below the active therapeutic levels. Moreover, dose- and time-dependent improvement by ABT-925 of cognitive impairment associated with schizophrenia has been recently reported (Abbs et al. 2012).

Hence, the present clinical experience with D_3_ antagonists in schizophrenia is inconclusive because the doses tested are supposed to be insufficient to reach a clinically active level in the two clinical studies reported so far. This raises an important question to be addressed: which is the minimal level of D_3_ receptor occupancy to be reached for obtaining an antipsychotic effect? Indeed, there is no clinical study linking D_3_ receptor occupancy and efficacy, so that the question has to be answered on the basis of indirect evidence. A comparison of the affinities of antipsychotic drugs at recombinant D_2_ and D_3_ receptors indicates that these compounds generally show some, but very limited, preference for the D_2_ receptor (Sokoloff et al. 1992; Sokoloff 2006). Hence, it can be assumed that 40–60 % D_3_ receptor occupancy occurs during antipsychotic treatments, resulting in 70–80 % D_2_ receptor occupancy, with D_2_ receptor occupancy greater than 80 % resulting in extrapyramidal side effect (Farde and Nordstrom 1993). Nevertheless, a PET study in baboons has suggested that D_3_ receptor occupancy by clozapine and haloperidol at clinically active doses is probably lower than the above figures (Girgis et al. 2011). However, since no safety concern has been raised with D_3_ antagonists until now, the doses to be tested in the clinics should be chosen to occupy a large fraction of D_3_ receptors (>80 %) to maximize the chance to get conclusive results, without compromising tolerability. The clinical experience with BP 897 and ABT-925 also indicates that the design of proof-of-concept studies with a novel mechanism should be supported by pharmacodynamic studies, such as PET studies when adequate tracers are available, in order to anticipate the clinically active doses. Furthermore, in drug development with new targets, the development of specific pharmacodynamic translational markers should be encouraged even before the initiation of a program.

## Conclusions 

The data collected so far indicate that the D_3_ receptor has the ability to exert a control on glutamatergic activity, by acting either directly on NMDA receptor signaling at glutamate synapses on terminals of pyramidal cells in the nucleus accumbens or indirectly through modulation of dopamine acting presynaptically on these cells in the prefrontal cortex. This property is unique, insofar as it is not shared by other dopamine receptor subtypes. Notably, the D_2_ receptor, which is still considered as the main target for antipsychotic drugs currently used in the clinics (Kapur and Mamo 2003), is localized in both the cortical and subcortical areas. In the striatum, its level of occupancy is correlated with the improvement of positive symptoms of schizophrenia. In the cortex, however, blocking the D_2_ receptor, as do current antipsychotic drugs, disrupts the complex D_1_–D_2_ control over the pyramidal cell/interneuron network and is certainly not optimal to treat schizophrenia, particularly negative symptoms and cognitive dysfunction. This is the reason why drug discovery is presently oriented toward the development of drugs acting at mGluR2/3 receptors or the glycine transporter (Kantrowitz and Javitt 2012) to correct glutamate dysfunctions in the prefrontal cortex. This approach needs to be consolidated by extensive clinical studies, replicating the encouraging findings of proof-of-concept studies. Also, evidence for long-term efficacy and safety of these drugs is lacking so far.

Selective D_3_ receptor blockade appears as a promising alternative to current D_2_/D_3_-based antipsychotics and a safe mean to modulate glutamate in schizophrenia. This pharmacological intervention may be able to correct both positive and negative symptoms of schizophrenia, as well as to improve cognitive deficits associated with the disease. Nevertheless, the D_3_ receptor hypothesis of schizophrenia is still mainly based on indirect or preclinical evidence. So far, it seems that the development of D_3_ compounds that have reached the clinics has been discontinued for unclear reasons except those related to the dose. The field of D_3_ drug discovery still appears active, and one can anticipate that suitable drugs used at doses anticipated to reach the target and occupy a large fraction of D_3_ receptors will be available to obtain conclusive clinical results.

## Abbreviations

AMPA: α-Amino-3-hydroxy-5-methyl-4-isoxazole-propionic acid
ANOVA: Analysis of variance
CaMKII: Ca^2+^/calmodulin-dependent protein kinase II
CGI: Clinical global impressions
[^11^C]-(+)-PHNO: [^11^C]-(+)-4-*N*-propyl-,3,4a,5,6,10b-hexahydro-2 *H*-naphtho[1,2-*b*][1,4]-oxazin-9-ol
GABA: γ-Aminobutyric acid
[^3^H]-7-OH-DPAT: [^3^H]-7-hydroxy-*N*,*N*-di-*n*-propyl-2-aminotetralin
[^125^I]-7-OH-PIPAT: [^125^I]-*R*-(+)-*trans*-7-hydroxy-2-(*N*-*n*-propyl-*N*-3’-iodo-2’-propenyl)aminotetralin
MK-801: Dizocilpine
NMDA: *N*-methyl-d-aspartate
PCP: Phencyclidine or *N*-(1-phenylcyclohexyl)-piperidine
PET: Positron emission tomography
PSD: Post-synaptic densities
PANSS: Positive and Negative Syndrome Scale
VGluT1: Vesicular glutamate transporter 1
VGluT2: Vesicular glutamate transporter 2
